# Intermodal all-optical pulse switching and frequency conversion using temporal reflection and refraction in multimode fibers

**DOI:** 10.1515/nanoph-2024-0653

**Published:** 2025-04-07

**Authors:** Alexis C. Sparapani, Yifan Sun, Fabio Mangini, Mario Ferraro, Govind P. Agrawal, Stefan Wabnitz

**Affiliations:** DIET Department, 9311 Sapienza University of Rome, Via Eudossiana 18, 00184, Rome, Italy; Service OPERA-Photonique, Université Libre de Bruxelles, 50 Avenue F. D. Roosevelt, B-1050, Brussels, Belgium; Department of Physics, University of Calabria, Via P. Bucci, Rende, 87036, Italy; The Institute of Optics, University of Rochester, Rochester, NY, 14627, USA

**Keywords:** optical fibers, temporal reflections, nonlinear optics, Kerr effect, optical switching, multimode fibers

## Abstract

In this work, we extend temporal reflection and refraction analogies from the case of singlemode optical fibers to multimode fibers. Specifically, we show that nonlinear multimode fibers provide novel degrees of freedom that permit us to control optical pulse interactions. We take advantage of the properties of pulses propagating in different modes, such as their group velocities, dispersion parameters, and effective mode areas, in the presence of the Kerr effect. Our study was carried out by numerically solving the multimode nonlinear Schrödinger equations for graded-index optical fibers. From this analysis, we find a useful tool to obtain novel all-optical switching and frequency conversion schemes in multimode fibers.

## 1 Introduction

All-optical switching schemes have been extensively investigated in recent years, because of their potential for ultrafast optical information processing [1], [2]. The performance of all-optical switches hinges on light-by-light control – e.g., the capability to control an optical pulse with another pulse at a different wavelength [3]. Such an all-optical control enables different phenomena such as ultrafast frequency conversion [4], tailored supercontinuum generation (SCG) [5], [6], [7], and optical signal processing [8], to name a few. The peculiar properties of light-by-light interactions have sparked an interest in studying analogies between all-optical gates [9], transistors [10], [11], [12], and multimode fiber based switches [13].

As in many other fields, nonlinear pulse propagation in optical fibers is widely used for establishing analogies with phenomena that occur in a totally different context. Among these, the optical analogy of the event horizon associated with a black hole stands out [14]: an intense pulse, e.g., a propagation-invariant wavepacket such as a soliton, locally increases the refractive index of the fiber along its own propagation path, because of the optical Kerr effect [15]. When a weaker probe pulse, typically dispersive wave (DW), propagates in the fiber at a different frequency, it may collide with the soliton because of their different group velocities. As a result, the DW encounters a temporal boundary, created by the soliton-induced refractive index change via the Kerr effect. After its collision with the soliton, the DW experiences a slight acceleration or deceleration in its group velocity, enabling it to either being “reflected” or “transmitted” through the light barrier. Such changes in group velocity result, via chromatic dispersion, from nonlinearity induced changes of the DW carrier frequency, so that the momentum of light is conserved [16]. These analogies have a close resemblance with the famous Snell’s law and the Fresnel coefficients, as discussed in Ref. [17].

Such spatiotemporal analogies have proved very useful for explaining fundamental optical interactions. Furthermore, they have been widely studied for applications such as signal processing, frequency conversion, optical switching [18], and pulse manipulation [19], [20].

As far as controllable parameters are concerned, several variables have been considered for controlling all-optical interactions. These include: the DW peak power [21], [22], the temporal width [23], wavelength, the time delay Δ*T* between the soliton and the DW [20], the temporal duration of the soliton [24], the peak power of the soliton [25], the order of the soliton [26] and its shape [27], and the initial chirp of the pump pulse [28], [29].

Furthermore, it is important to consider the influence of the medium where optical pulses interact. In the case of singlemode fibers, several studies have considered the role of the zero-dispersion wavelength (ZDW) [5], [30], the presence of multiple ZDWs in the case of photonic crystal fibers (PCF) [31], [32], and the existence of a zero-nonlinearity wavelength (ZNW) for doped PCFs [25]. Temporal reflection and refraction have also been studied for silicon [33] and photonic crystal waveguides [34].

Several all-optical control schemes have been considered for silica fibers, both theoretically and experimentally. Noteworthy among them is the experiment reported in Ref. [22], where temporal bouncing of a DW, trapped between two solitons, was observed. It was found that the DWs are frequency shifted in opposite directions upon successive collisions with the two solitons. Numerical simulations, based on solving the generalized nonlinear Schrödinger equation (GNLSE) [35], were in good agreement with the experimental data.

**Figure 1:**
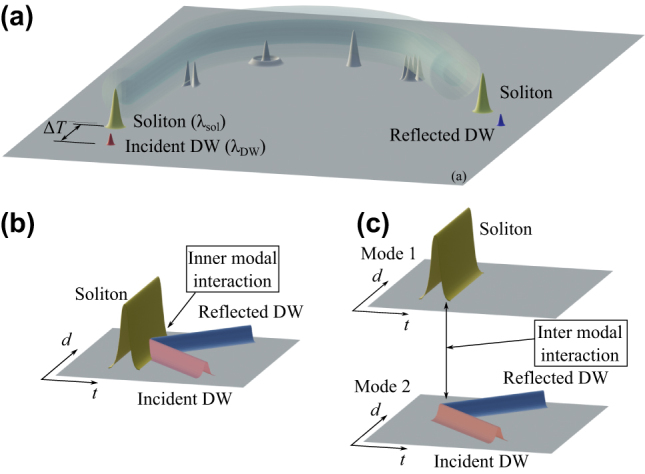
Schematic representation of a soliton-dispersive wave (DW) interaction in a multimode optical fiber. (a) At the input we inject a soliton at *λ*_sol_ and a DW pulse at *λ*_DW_, delayed Δ*T*. At the output, one obtains a transmitted and a reflected DW pulse, both traveling at different velocities from the soliton, after the pulse interaction process. Along the fiber we schematically represent the different fiber modes. (b) Temporal reflection for the singlemode case, showing the intra-modal interaction, and (c) for soliton and DW propagating in different modes.

Multimode fibers (MMFs) support the formation of multimode solitons [36], which have drawn significant interest, particularly in the case of graded-index (GRIN) MMFs. Studies on multimode solitons in MMFs have investigated phenomena such as walk-off solitons [37], singlemode spatiotemporal soliton attractors [38], soliton collisions [39], dispersive radiation sideband generation [40], [41], and multimode soliton dynamics in step-index multimode fibers [42], [43]. To our knowledge, all prior studies on all-optical control in fiber systems have been limited to single spatial mode configurations. In this context, exploring the interactions between multimode solitons and multimode dispersive waves (DWs) appears as a compelling subject for further investigations. In this work, we provide the first step in this direction, by theoretically studying optical information processing in MMFs based on soliton-DW interactions in a GRIN fiber. More specifically, we leverage the modal degrees of freedom by allowing the DW and the soliton to propagate in separate modes within the GRIN fiber. This approach still permits us to control the reflection and refraction of the DW at a temporal barrier, as shown schematically in Figure 1. At the same time, our scheme adds new control parameters for the nonlinear pulse interaction and frequency conversion process, such as the specific modes that carry the soliton and the DW in the GRIN fiber. Moreover, the multimode approach permits us to take advantage of the difference in linear group velocities of the DW and the soliton that propagate in two different modes. Our numerical results are based on solving the relevant multimode nonlinear Schrödinger equation (MMNLSE) [44]. A particular noteworthy case involves the temporal barrier which is created by the propagation of a multimode soliton. In this work we restrict our attention to soliton-DW interactions in GRIN MMFs. A similar phenomenology is expected for the case of step-index MMFs. However, it is worth mentioning that the generation of a multimode soliton in a step-index fiber is more challenging than in GRIN fibers: because of their much larger modal dispersion, higher intensities are required to trap multiple modes via nonlinearity (see Ref. [43] for details).

The manuscript is organized as follows. We present in Section 2 the results obtained by propagating the soliton and DW in two different modes of a GRIN fiber, and exploiting intermodal interaction between them. Next, we study in Section 3 the influence of different modes by computing the fraction of pulse energy that is reflected or transmitted through the temporal barrier. In Section 4, we propose a novel switching scheme that is unique to MMFs: it is based on the interaction of a DW with a multimode soliton. In Section 5, we discuss DW reflection from multimode  solitons. We conclude in Section 6 by summarizing our main results.

## 2 Temporal reflection in multimode fibers

The nonlinear Schrödinger equation (NLSE) is commonly used to describe pulse dynamics in singlemode fibers or waveguides [35]. Generally speaking, solving the nonlinear wave equation permits us to model a variety of phenomena, including the temporal analogs of Fabry–Perot resonators [45], lasers [46] and optical event horizons [15]. Here, our objective is to explore the temporal analogies of reflection and refraction in the case of MMFs. For this goal, we employ the MMNLSE [36], [44], [47], [48], which has been extensively and successfully applied to study nonlinear pulse dynamics in MMFs. Specifically, for the present analysis we can write the MMNLSE in the following simplified form

(1) $$\begin{array}{cc} \partial_{z}A_{p}\left(z,t\right) & =i\delta \beta_{0}^{\left(p\right)}A_{p}-\delta \beta_{1}^{\left(p\right)}\partial_{t}A_{p}\\ & \;+\overset{N_{d}}{\underset{m=2}{\sum }}i^{m+1}\frac{\beta_{m}^{\left(p\right)}}{m!}\partial_{t}^{m}A_{p}\\ & \;+i\frac{n_{2}\omega_{0}}{c}\overset{M}{\underset{l,m,n}{\sum }}S_{\mathrm{plmn}}A_{l}A_{m}A_{n}^{*}, \end{array}$$

where *A*_*p*_ is the field envelope of mode *p* (*p* = 1 indicates the fundamental mode), $\beta_{m}^{\left(p\right)}$ is the *m*-th order dispersion of mode *p*, $\delta \beta_{0,1}^{\left(p\right)}=\beta_{0,1}^{\left(p\right)}-\beta_{0,1}^{\left(1\right)}$ , *n*_2_ is the nonlinear index, 2*πc*/*ω*_0_ is the center wavelength, and {*S*_*plmn*_} are the nonlinear mode coupling coefficients (i.e., the spatial mode overlap). In short, the multimode pulse evolution upon propagation is given by dispersion effects [the first three terms on the right side of Eq. (1)] plus Kerr nonlinearity [last term in Eq. (1)]. For the sake of simplicity, here we did not consider the presence of stimulated Raman scattering (SRS). Our main focus is to investigate the temporal analogies of reflection and refraction due to the interaction of soliton-DW in the presence of the instantaneous Kerr nonlinearity only. For the case of singlemode fibers, such a study was earlier carried out, e.g., in Refs. [3], [25]: now we extend the treatment to pulses propagating in different modes. The justification for neglecting SRS is that its presence, whose most notable manifestation is the soliton self-frequency shift, does not affect our main findings. In our modeling, we included dispersive terms up to the third-order, i.e., *β*_3_, and we set *β*_*m*_ = 0, where *m* > 3. Nevertheless, we plan to investigate the possible influence of SRS in a subsequent study. We may anticipate that SRS will play a significant role whenever the wavelength difference between pulses is close to 100 nm, so that it induces a power transfer from the short wavelength (pump) to the long-wavelength (Stokes) pulse. Finally, our analysis was carried out considering up to *M* = 15 modes. However, in most cases shown in this paper, we performed simulations by considering two modes only, i.e., by launching pulses in only two modes and leaving the others without excitation.

Let us consider a 5 m-long GRIN fiber with a ZDW at *λ*_ZDW_ = 1345 nm. Moreover, the core-radius of the fiber is *R* = 25 *μ*m, and the core-cladding relative index difference is Δ = 0.0137. The pump and probe pulses are launched in separate modes, with different carrier wavelengths. As a result, the pulses propagate with different speeds because of both modal and chromatic dispersion. More specifically, we consider a 85-fs pump pulse at *λ*_sol_ = 1550 nm, which forms a first-order soliton propagating in the fundamental Laguerre–Gaussian (LG) mode, the LG_01_ mode. The wavelength of the DW or 300-fs-wide probe pulse is *λ*_DW_ = 1190 nm: this pulse propagates in LG_11a_ mode, with an initial advance of Δ*T* = 0.75 ps, i.e., it leads the soliton by 0.75 ps. Note that the wavelengths of two pulses lie on opposite sides of the ZDW, i.e., they propagate in different dispersion regimes of the GRIN fiber.

**Figure 2:**
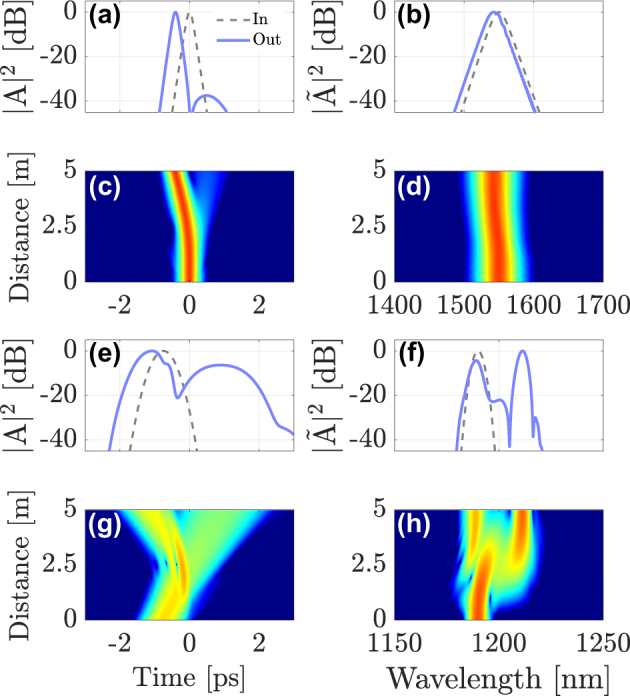
Intermodal interaction between a first-order soliton in the fundamental mode LG_01_, launched at *λ*_sol_ = 1550  nm and a DW pulse in mode LG_11a_, propagating at *λ*_DW_ = 1190 nm. (a)–(b) intensity profiles of the soliton; (c)–(d) soliton evolution; (e)–(f) input and output shapes and spectra of the DW; (g)–(h) evolution of the DW pulse along the fiber.

Figure 2 shows the results of our numerical simulations. In Figure 2a and b we show the input (dashed gray line) and output (solid blue line) intensity profiles of the soliton in the time and frequency domains, respectively. The temporal and spectral soliton evolution along the GRIN fiber is shown by using color maps in Figure 2c and d. The panels in Figure 2e–h show the corresponding evolutions for the DW probe.

As seen in Figure 2, the two pulses collide at about 2.5 m in the GRIN fiber, because of their different speeds, and they interact through cross-phase modulation (XPM). Intermodal XPM introduces a mutual change in the refractive index seen by the two pulses: the weak probe pulse is strongly affected by XPM, because of the presence of the intense soliton in the fundamental mode. Similar to the singlemode case, the incident DW splits into a reflected and a refracted pulse, which propagate with different speeds, as is shown in Figure 2e–g. In the frequency domain, as a result of hitting the refractive index discontinuity, the reflected pulse is considerably red-shifted with respect to the incident pulse, whereas the transmitted pulse exhibits a slight blue-shift. This is evident from the probe spectra in Figure 2f and h.

The probe pulse also affects the pump soliton, if its energy is not negligible compared to that of the pump. As seen in Figure 2, the soliton trajectory bends after the collision because of a small change in its own speed, which is induced by the probe together with a weak frequency shift. The reflected and transmitted frequencies can be derived from the dispersion relation and using the conservation of momentum, for details see Ref. [16].

**Figure 3:**
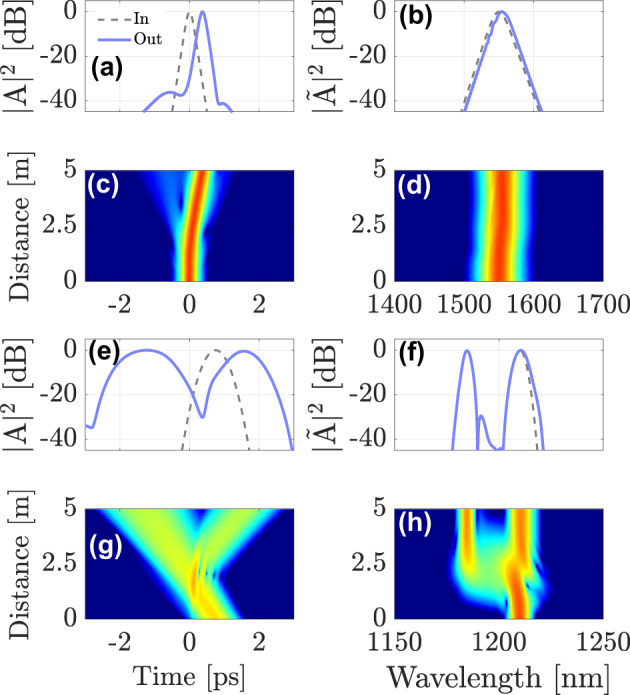
Intermodal interaction between a first-order soliton in the fundamental mode LG_01_, launched at *λ*_sol_ = 1550 nm and a DW carried by mode LG_11a_, propagating at *λ*_DW_ = 1210 nm. (a)–(b) intensity profiles of the soliton; (c)–(d) soliton evolution; (e)–(f) input and output shapes and spectra; (g)–(h) evolution of DW probe pulse.

In Figure 3 we show another example of temporal reflection. For this case, we changed the DW wavelength to *λ*_DW_ = 1210 nm: as can be seen, in this case the DW propagates faster than the soliton. Therefore, we adjusted its initial temporal delay to ensure that it still collides with the soliton in the middle of the GRIN fiber. Similar to the singlemode case, one can control the XPM interaction in the multimode case by changing the wavelength and the initial delay of probe pulses. As seen in Figure 3, when the probe reaches the temporal barrier, the spectrum of the reflected pulse is shifted towards the blue side of the input spectrum. That is, the sign of the reflected pulse’s wavelength shift is opposite to that in Figure 2. This example shows that both red and blue wavelength shifts can be obtained, by simply changing the input parameters of the probe pulses. Equivalently, such a change of sign of the frequency shift due to the collision can also be obtained by changing the carrier frequency of the soliton control pulse.

## 3 Mode dependence of temporal reflection

Multimode fibers provide a new degree of freedom, consisting in the possibility of varying the mode combination of pump and probe pulses. Since the XPM interaction depends on the modal overlap, the efficiency of the reflection process will not be the same for all combinations. In this section, we investigate this property by using four different combinations. We suppose that pump pulses are always launched into the fundamental mode of the fiber, while the probe pulses are launched in different individual modes.

**Figure 4:**
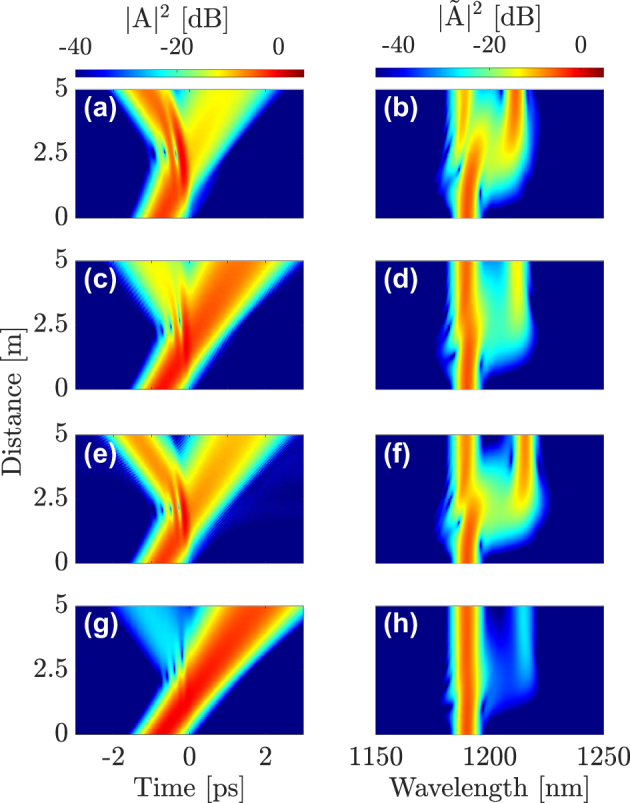
Interaction of a first-order soliton in the fundamental mode LG_01_ along with a DW at *λ*_DW_ = 1190 nm in the (a)–(b) LG_11a_, (c)–(d) LG_21a_, (e)–(f) LG_02_ or (g)–(h) LG_31b_ mode, respectively.

Figure 4 shows the simulation results for probe pulses propagating in four different modes: LG_11a_ (a)-(b), LG_21a_ (c)-(d), LG_02_ (e)-(f), and LG_31b_ (g)-(h). Each row shows the temporal (a, c, e and g) and spectral (b, d, f and h) evolution along a 5-m-long GRIN fiber. These results expose the influence of the mode choice on the XPM interaction. Even though temporal reflection occurs in all cases, its efficiency depends on the mode in which the probe is initially launched. Considerable reflection occurs in the first and the third cases, but the efficiency drops considerably in the second case, and even more in the fourth case, where the DW pulse is nearly completely transmitted.

**Figure 5:**
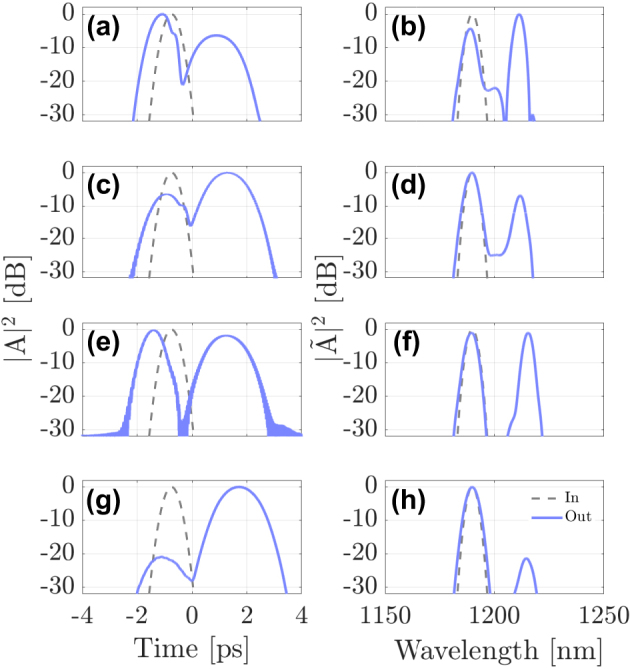
Input (dashed-gray line) and output (solid-blue line) of the DW at *λ*_DW_ = 1190 nm in the LG_11a_ (a)–(b), LG_21a_ (c)–(d) and LG_02_ (e)–(f) and LG_31b_ (g)–(h) in time and frequency domain, respectively.

**Figure 6:**
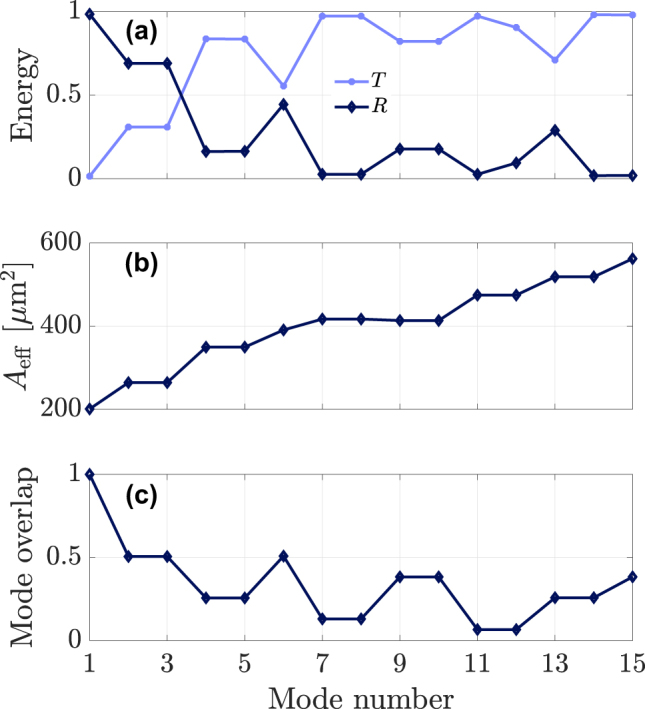
Transmitted, *T*, (solid light-blue line) and reflected, *R*, (dotted blue line) pulses after the collision with the fundamental soliton depending on the initial mode of the DW (a). Effective area (b) and normalized mode overlap of each mode (c).

To elucidate the modal dependence of the soliton-DW interaction more clearly, we compare in Figure 5 the input and output shapes and spectra of the probe pulse in the four cases shown in Figure 4. Figure 5 highlights the differences between these cases more clearly. For the case shown in Figure 4a and b (DW in mode LG_11a_), the probe splits into its transmitted and reflected parts, with more than 60 % reflection efficiency (see Figure 6a). However, when the probe is launched into the LG_21a_ mode (c and d panels of Figure 4), the reflection efficiency is reduced considerably, down to about 10 %. In the (e) and (f) panels of Figure 4, the probe pulse is launched into the LG_02_ mode, and the reflection efficiency reaches up to 50 %. A remarkable feature is that the spectral shift of the reflected pulse is larger in this case when compared to the first case of Figure 5a and b. In the last case, see panels (g) and (h), the probe is launched into the LG_31b_ mode, and the reflection efficiency drops down by more than 20 dB. Since the reflected pulse exhibits a significant frequency shift after the interaction, reflection efficiency has the meaning of a frequency conversion efficiency.

We carried out a systematic investigation of the mode dependence of the reflection efficiency of the probe when coupled to each of the 15 different modes of the GRIN fiber, while keeping the soliton in the fundamental mode. In each case, we numerically computed the fraction of the DW pulse’s energy in the transmitted and reflected pulses. Moreover, we also computed the modal effective mode areas [36]

(2) $$A_{\text{eff}}^{\left(p\right)}=\frac{{\left(\iint |F_{p}\left(x,y\right){|}^{2}dx\;dy\right)}^{2}}{\iint |F_{p}\left(x,y\right){|}^{4}dx\;dy},$$

and the modal overlap coefficients

(3) $$C_{}^{\left(p,q\right)}=\frac{\iint |F_{p}\left(x,y\right){|}^{2}|F_{q}\left(x,y\right){|}^{2}dx\;dy}{\iint |F_{p}\left(x,y\right){|}^{2}dx\;dy\iint |F_{q}\left(x,y\right){|}^{2}dx\;dy},$$

of the different combinations of modes profiles, i.e. *F*_*p*_(*x*, *y*) and *F*_*q*_(*x*, *y*).

Figure 6a shows the mode dependence of energy fraction in the transmitted (*T*) and reflected (*R*) pulses, together with the modal effective mode areas (Figure 6b) and modal overlap coefficients (Figure 6c). As can be seen, the efficiency of the probe reflection process decreases when the effective mode area *A*_eff_^(p)^ grows larger. A stricter correlation is observed between the temporal reflection efficiency and the modal overlap coefficient *C* ^(p,1)^/*C* ^(1,1)^, normalized to the fundamental mode. For example, the modal overlap of the fundamental mode locally increases for mode 6, which corresponds to the radially symmetric LG_02_ mode, and this leads to a corresponding peak in the efficiency of temporal reflection.

## 4 Impact of fiber dispersion

So far, pump and probe pulses were propagating in different dispersion regimes of the GRIN fiber; the soliton in the anomalous dispersion region and the probe in the normal dispersion region. This was done to ensure that the difference in their group velocities was relatively small [15]. While this arrangement is necessary for singlemode fibers, it is not required for multimode fibers, where we can take advantage of the different group velocities associated with fiber modes. Therefore, we now consider the case where both pump and probe pulses propagate in the anomalous dispersion region, while their wavelength difference is relatively small. The pump pulse is intense enough to form a soliton, but the probe pulse propagates as a DW.

**Figure 7:**
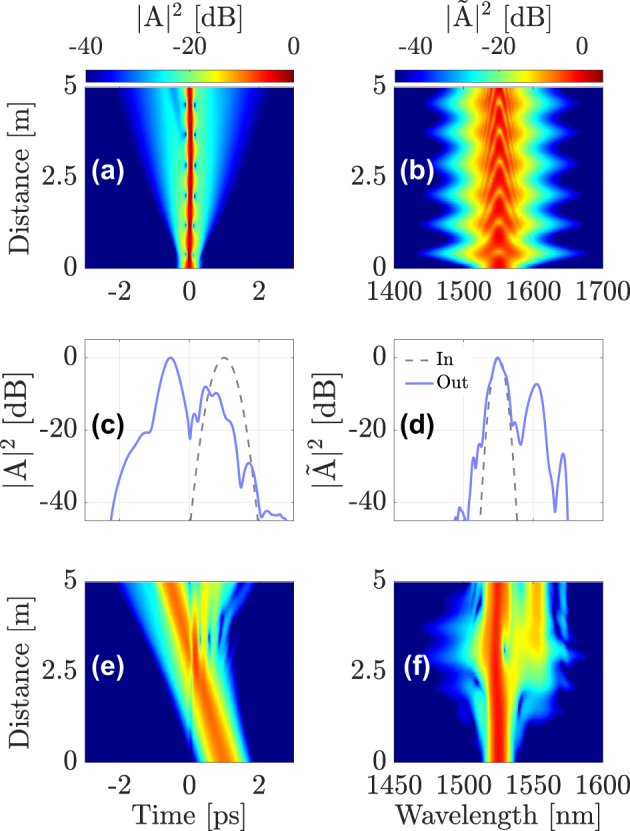
Interaction of a soliton in the fundamental mode at *λ*_sol_ = 1550 nm (a,b) with a dispersive wave at *λ*_DW_ = 1525 nm in mode LG_21a_; output intensity profile of the DW in time domain (c) and frequency domain (d), respectively; DW evolution in temporal (e) and frequency (f) domains.

Figure 7 shows the results of a numerical simulation for *λ*_sol_ = 1550 nm and *λ*_DW_ = 1525 nm, with the two pulses separated by Δ*T* = 1 ps. The peak power of the pump pulse corresponds to the soliton order *N* = 1.5. The pump is again launched into the fundamental mode of the fiber, and the probe into the LG_21a_ mode. Figure 7a and b show the pump evolution, and Figure 7e and f show the probe evolution over 5 m of a GRIN fiber. Figure 7c and d compare the input and output profiles of the probe in the time and spectral domains.

As can be seen in Figure 7, XPM-induced temporal reflection still occurs when the two pulses collide in the middle of the fiber, and the spectrum of the reflected pulse shifts towards the red side.

**Figure 8:**
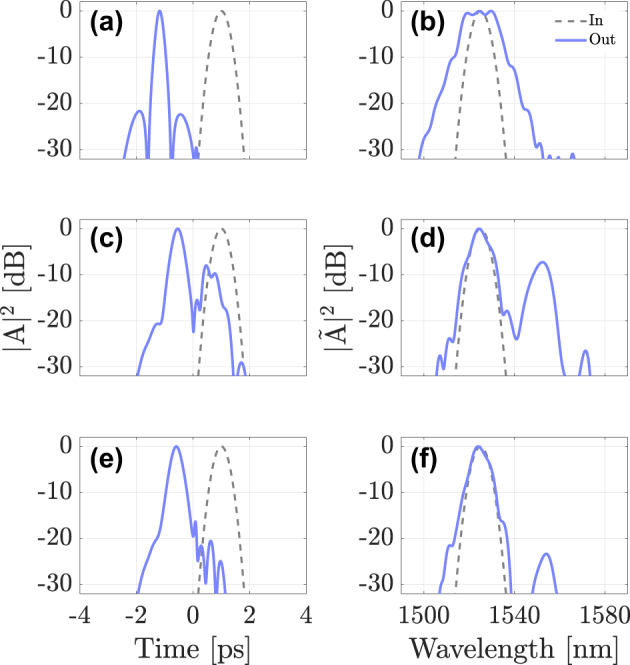
Intensity profiles for a DW injected at *λ*_DW_ = 1525 nm, for three different cases: DW coupled to mode LG_11a_ (a,b), mode LG_21a_ (c,d) or mode LG_02_ (e,f), at the fiber input (dashed grey lines) and output (solid blue lines); the soliton propagates in the fundamental mode at *λ*_sol_ = 1550 nm.

Similar to the case where the DW propagates in the normal dispersion regime, the reflection efficiency is expected to depend on the fiber mode into which the probe is coupled. Figure 8 shows, in the time (left column) and frequency (right column) domains, the cases where the probe is launched into either the LG_11a_ mode (panels) (a) and (b)), the LG_21a_ mode (panels (c) and (d)), or the LG_02_ mode (panels (e) and (f)). As before, temporal reflection is strongly dependent on the mode in which the probe is traveling. In the first and third cases, the probe pulse is mostly transmitted, but considerable reflection of the probe occurs in the second case. Therefore, by exploiting the spatial degree of freedom one may observe efficient temporal reflection and refraction with carrier wavelengths of the two interacting pulses that are separated by a few nanometers only. This is an important advantage of our multimodal approach with respect to the singlemode case, which requires pulses that propagate in opposite dispersion regimes. Specifically, the center wavelengths of all interacting pulses may fit in the same telecom window (e.g., the C-band). This means that our approach may be used as a means for all-optical frequency conversion and routing among different wavelength-division-multiplexed channels*.*

## 5 Reflection from multimode solitons

So far, we considered cases where the pump pulse is provided by a first-order soliton propagating in the fundamental mode of the GRIN fiber, that is, a singlemode soliton. As well known, GRIN fibers support the formation of multimode solitons, where the soliton is composed of several low-order modes [36]. An interesting question is: what happens when such a multimode soliton is used for the temporal reflection of a probe pulse?

**Figure 9:**
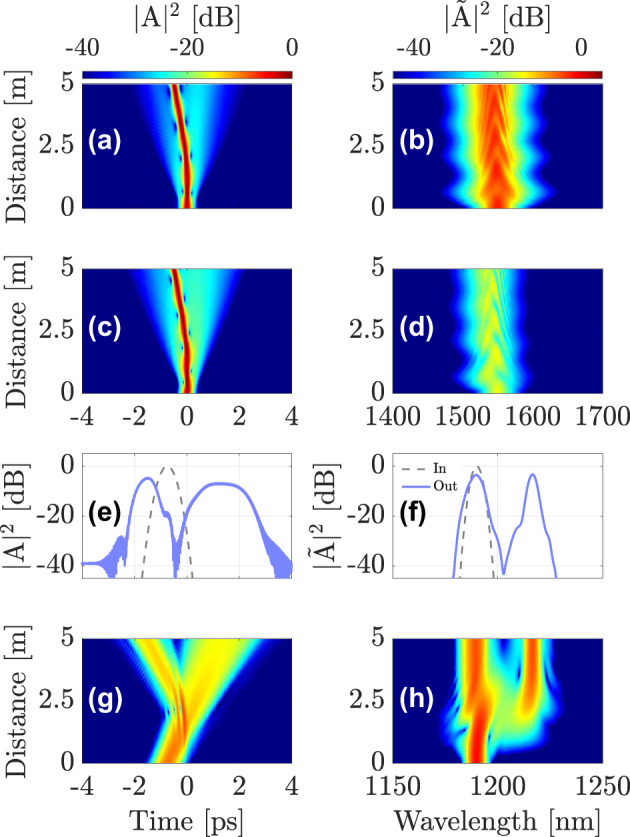
Interaction of a multimode soliton (with 80 % of its power in the fundamental mode LG_01_ (panels (a) and (b)) and 20 % in mode LG_11a_ (panels (c) and (d)) with a DW at *λ*_DW_ = 1190 nm propagating in mode LG_21a_: panels (e) and (f) show input (dashed curves) and output (solid curves) intensity profiles of the DW in the temporal and spectral domain, respectively; panels (g) and (h) show the corresponding evolutions of the DW along the fiber.

Figure 9 shows a case where a 1550nm pump pulse propagates as a multimode soliton, with 80 % of its energy in the fundamental mode and the remaining 20 % in the LG_11a_ mode. The 1190nm probe pulse propagates in the LG_21a_ mode of the GRIN fiber, that is in the normal-dispersion region. Panels (a) and (b) show the evolution of the multimode soliton component in the fundamental mode, and panels (c) and (d) of its component in the LG_11a_ mode. The DW probe evolution is illustrated in panel (g) and (h), while panels (e) and (f) compare the probe input and output profiles, in time and frequency, respectively. From these results we can see that a multimode soliton may also be used as a temporal barrier, which forces the DW probe to split into a reflected and transmitted pulse upon their collision.

## 6 Conclusions

In this work, we carried out a numerical analysis of temporal reflection and refraction effects in multimode GRIN fibers. We focused mainly on the case where the pump and probe pulses propagate in different modes of the GRIN fiber, and solved the multimode nonlinear Schrödinger equations that include effects associated with the Kerr nonlinearity of the fiber. Based on the different overlaps and effective areas of different modes, we explored the new degrees of freedom which enable the control of the efficiency of temporal reflection. Specifically, we have shown that the reflection efficiency, along with its associated frequency shift of the DW pulse, depends on the choice of the modes for the pump and probe pulses, and has a direct correlation with the effective modal overlap coefficient.

We also investigated a different pulse interaction scheme that is peculiar to multimode fibers, which takes advantage of the different group velocities of the modes. Modal dispersion allows for the pump and probe pulses to be both launched in the anomalous dispersion region of the fiber, with a relatively small difference in their wavelengths. We observed several new properties that may be beneficial for all-optical frequency switching of wavelength-division-multiplexed channels in optical transmission systems operating in the third telecom window. Since GRIN fibers support the formation of multimode solitons, composed of several low-order modes, we also demonstrated the possibility of using such a soliton for the temporal reflection and frequency conversion of a weak probe pulse. Our results indicate that the phenomenon of temporal reflection in multimode fibers provides an opportunity for exploring novel all-optical switching and frequency conversion schemes.
